# Effect of UV Light on the Barrier Performance of Aluminum Powder/Epoxy Coating

**DOI:** 10.3390/polym14122405

**Published:** 2022-06-14

**Authors:** Zhe Zhai, Hang Li, Lajun Feng, Feng Li

**Affiliations:** 1 School of Materials Science and Engineering, Xi’an University of Technology, Xi’an 710048, China; lihang0512@163.com (H.L.); fenglajun@163.com (L.F.); fli02@126.com (F.L.); 2 Xi’an Peak Xuan Kai New Material Co., Ltd., Xi’an 710024, China

**Keywords:** epoxy coating, UV aging, aluminum powder, EIS

## Abstract

Spherical aluminum powder was added to an epoxy composite coating in order to improve its protection performance for metal materials. The strength of the coating before and after UV (Ultraviolet Light) aging, its yellowing value, and its barrier properties were tested to explore the influence of UV light on the barrier performance of aluminum powder/epoxy coating. The results show that the addition of the aluminum powder enhanced the strength of the epoxy coating and reflected environmental UV light. This improved the resistance of the coating to UV aging and prolonged its service life. The composite prepared with 6 wt.% aluminum power exhibited the highest strength values before and after aging: 64.5 MPa and 58.5 MPa, respectively. After aging, the strength loss rate of this epoxy coating was 9.3%, and its yellowing value was +1.43.

## 1. Introduction

Metal failure caused by corrosion has led to huge economic losses in society and aroused widespread concern in recent years [1,2]. Protective coatings are one of the main strategies used to prevent metal corrosion [3,4]. Among the known protective coatings, epoxy resin is commonly used due to its good adhesion, heat resistance, and chemical resistance. However, pure epoxy coatings are prone to being affected by light, heat, water, oxygen, and other environmental factors. These factors degrade the performance of coatings and reduce their useful life [5,6]. In particular, under UV irradiation, epoxy resins exhibit yellowing, brittleness, cracking, and other performance degradation phenomena, which seriously affect their protective effect on metal substrates [7,8].

The traditional methods for improving the anti-UV aging resistance of polymer materials primarily involve the addition of ultraviolet absorbers and light stabilizers. However, ultraviolet absorbers and light stabilizers are organic materials. With increasing UV exposure time, the protective performance of these materials gradually decreases until they fail [9,10]. Compared with organic additives, inorganic nanomaterials such as nano-ZnO and nano-TiO_2_ show good performance due to their strong UV shielding properties, non-toxicity, high stability, lack of migration, and other excellent characteristics [11,12]. However, under UV light, some transition electrons and uncombined holes in inorganic nanoparticles with semiconductor properties can react with adsorbed water or other substances on nanoparticle surfaces to generate hydroxyl radicals and reactive oxygen radicals [13]. These highly reactive radicals can damage the molecular structure of matrix resins, leading to their degradation. Hence, when using inorganic nanoparticles to enhance the anti-UV aging resistance of resins, a free radical trapping agent is required to capture the resulting active free radicals.

Metal powders can also be added to resins to take advantage of their excellent ultraviolet reflection characteristics. These metal powder additives can be used without any other additives [14,15,16]. Among the potential metal powders, aluminum powder exhibits the best UV reflectivity: it can reflect more than 60% of the UV radiation in sunlight. Therefore, adding an appropriate amount of aluminum powder to epoxy resin can prevent the aging phenomenon caused by UV irradiation. In turn, this can improve the UV aging resistance of the epoxy resin material and extend its service life [13,17]. Our team added the home-made alumina-free powder particles to the epoxy, which can effectively improve the barrier performance of the coating to the water molecules in the environment, and effectively reduce the deterioration of mechanical properties by the UV light [18,19]. In this work, a varying amount of spherical aluminum powder was added to an epoxy resin, using the EIS test to explore the influence of ultraviolet light on the barrier properties of aluminum powder/epoxy resin composite coating.

## 2. Experimental

### 2.1. Materials and Sample Preparation

**Figure 1:**
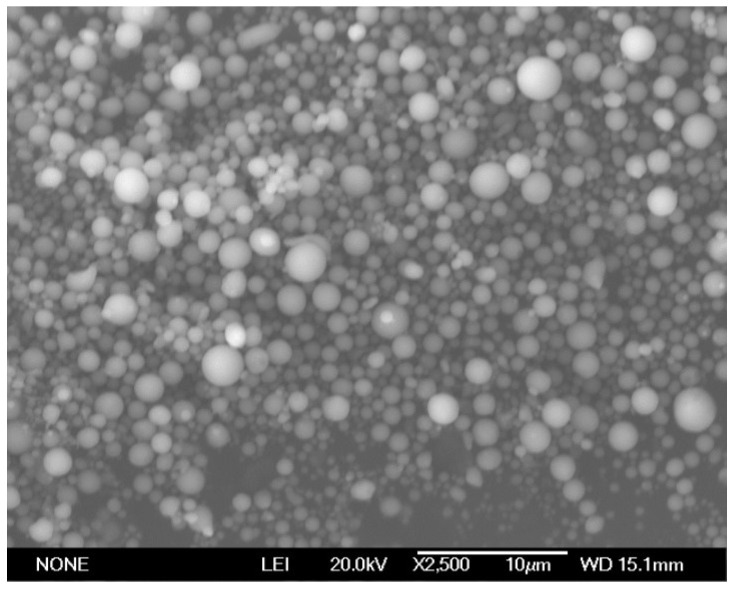
The SEM photograph of the aluminum powder.

Bisphenol-A epoxy resin with an epoxy equivalent of 210–244 g/eq was provided by Shandong Tianmao Chemical Co., Ltd. (Jinan, Shandong, China). The curing agent diethylene triamine (NH(CH_2_CH_2_NH_2_)_2_) was obtained from Wuxi Resin Co., Ltd. (Wuxi, Jiangsu, China). The spherical aluminum powder with a diameter of about 0.1–1 µm was provided by Beijing Decedao Gold Technology Co., Ltd. (Beijing, China). The purity of the aluminum powder was 99.5%. Figure 1 is the SEM picture of the aluminum powder. The metal substrate was Q235 steel, and its chemical composition is displayed in Table 1.

### 2.2. Preparation of Composite Material

First, the spherical aluminum powder was added to an isopropyl alcohol solution for ball milling to remove the organics on its surface. The ball milling time was 10 min, the milling speed was 400 r/min, and the ratio of ball:material ratio was 1:10. After the ball milling, an aluminum powder/isopropyl alcohol mixed solution was obtained. This mixture was washed with fresh isopropanol to remove the organics dissolved in the isopropanol during the ball grinding process. Next, the cleaned aluminum powder/isopropyl alcohol mixture was heated to 80 °C to volatilize the solvent. When the concentration of aluminum powder in the mixture reached about 80 wt.%, the epoxy resin was added to the mixture, which was evenly mixed and heated at 80 °C until the isopropyl alcohol was completely volatilized to obtain the aluminum powder/epoxy resin mixture. Next, the aluminum powder/epoxy resin mixture was treated in a vacuum oven to remove air bubbles at room temperature. A curing agent was then added to the aluminum powder/epoxy resin mixture with an epoxy resin to curing agent mass ratio of 100:8. Throughout this process, the mixture was slowly stirred with a magnetic stirrer to avoid the introduction of air into the resin. After the resin was completely mixed with the curing agent, the resulting mixture was quickly poured into a silicone mold to prepare block samples or coated on the Q235 steel surface with a coating machine to prepare a coating with a thickness of 20 ± 5 μm. This epoxy composite material was held at room temperature (25 °C) for 12 h, then cured in an oven at 80 °C for 4 h. After cooling, the epoxy was allowed to cool to room temperature in the oven.

### 2.3. Tensile Strength

A universal material testing machine was used to measure the tensile strength of the aluminum/epoxy resin block material. The test specimen had dimensions of 80 mm × 10 mm × 5 mm and the tensile rate was 0.5 mm/min. Each sample group was measured three times, and average values were used for analysis.

The formula for the loss of tensile strength after UV aging is as follows:

(1) $$\eta \%=\frac{\sigma_{0}-\sigma }{\sigma_{0}}\times 100\%,$$

where *σ*_0_ is the strength of the sample before UV aging, *σ* is the strength of the sample after UV aging, and *η*% is the strength loss rate.

### 2.4. Accelerated UV Aging

The samples were placed in an accelerated UV aging box to undergo 100 days of prolonged UV irradiation. After the accelerated aging process, the aged samples were characterized by their tensile strength, surface yellowing, and electrochemical impedance spectroscopy (EIS).

### 2.5. Yellowing Value

The color tristimulus values (X, Y, Z) of the composite epoxy coatings before and after UV aging were tested using a spectrodensitometer (X-Rite Inc., Grandville, MI, USA). Based on the measured values, the yellow color index (YI) and the yellowing value (ΔYI) were calculated by using the following equations:

(2) $$\mathrm{YI}=\frac{100(1.28X-1.06Z)}{Y}$$

(3) $$\Delta \mathrm{YI}=\mathrm{YI}-{\mathrm{YI}}_{0}$$

where X, Y, and Z are the color tristimulus values, YI_0_ is the yellow color index of the composite before UV aging, and YI is the yellow color index of the composite after UV aging. ΔYI was used to characterize the speed of yellowing and aging.

### 2.6. EIS Testing

EIS measurements were performed with an electrochemical workstation (CS350H, Wuhan Koster Company, Wuhan, Hubei, China). A three-electrode system composed of a platinum electrode (the auxiliary electrode), a saturated calomel electrode (the reference electrode), and the working electrode was used for testing. The working electrode was a Q235 steel sample coated with the epoxy resin composite coating. The electrolyte solution was a 3.5 wt.% NaCl solution. The working area of the working electrode was 1 cm^2^, and a frequency range of 10 mHz to 100 kHz was used. Before each EIS test, the open circuit potential (OCP) was run for 1200 s to obtain a stable test environment. To avoid allowing the solution concentration to affect the test results, the electrolyte solution was regularly replaced to keep its composition stable. The capacitance and resistance of the coating were obtained by fitting the measured impedance data.

## 3. Results and Discussion

### 3.1. Tensile Strength 

**Figure 2:**
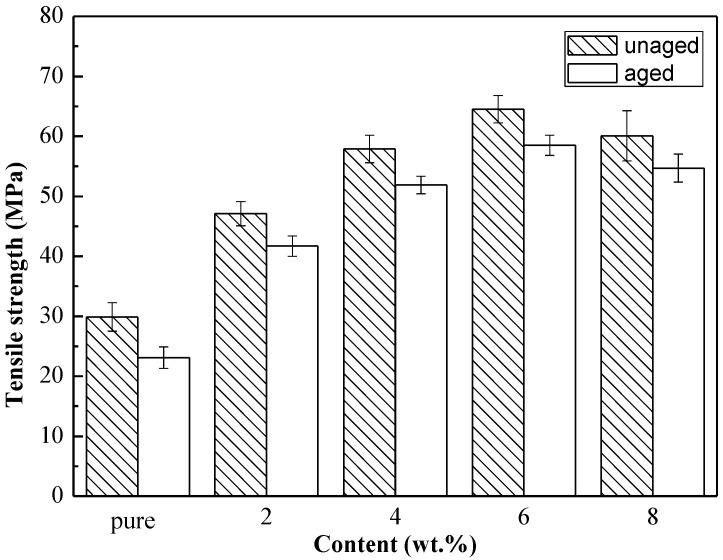
Tensile strength of pure epoxy and composites with different spherical aluminum powder content.

**Figure 3:**
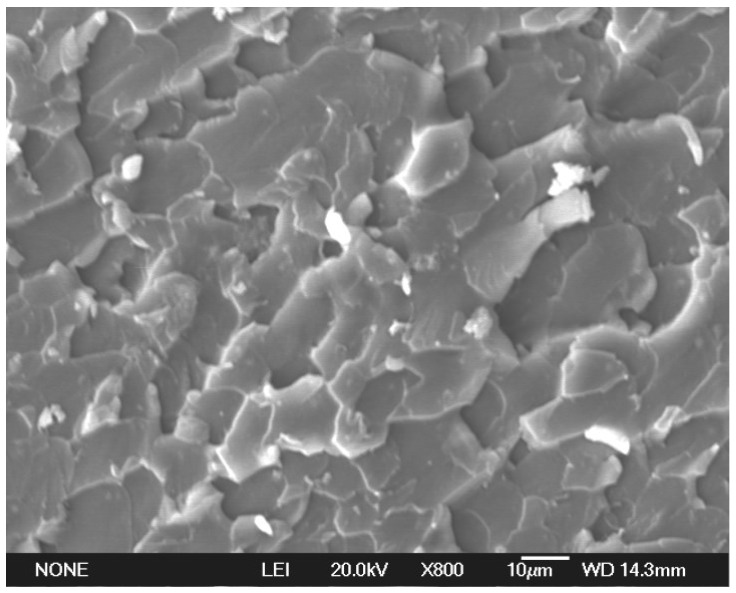
The stretch section of the composite with 6 wt.% aluminum powder.

The tensile strengths of the pure epoxy and spherical aluminum powder/epoxy composite before and after UV aging are shown in Figure 2. Figure 3 is the stretch section of the composite with 6 wt.% aluminum powder. The morphology likes some homogeneous fish scales. It shows that the spherical aluminum powder was evenly dispersed in the resin, and effectively improves the strength.

The addition of spherical aluminum powder effectively improved the strength of the unaged epoxy resin. In addition, with increasing spherical aluminum powder content, the strength of the composite material showed a trend of first increasing and then decreasing. A maximum tensile strength of 64.5 ± 2.5 MPa was achieved with 6 wt.% spherical aluminum powder. This was potentially because the microcracks inside the epoxy material encountered aluminum particles in the process of propagation. Thus, the microcracks were blocked by the rigid aluminum particles. Under the external load, the blocked crack tip underwent deflection or even forked to form a secondary crack. However, both crack deflection and the generation of secondary cracks required the absorption of more energy, which effectively consumed the energy of the original crack [20,21].

After 100 days of UV aging, the tensile strengths of all the composites were reduced. However, the strengths of the aged composite were still higher than that of the unaged pure epoxy. The tensile strength loss rates of the composite materials after UV aging were calculated according to Equation (1), as shown in Table 2. The tensile strength loss rate of these composite materials after UV aging gradually decreased with increasing spherical aluminum powder content. Thus, high spherical aluminum powder led to the formation of a composite epoxy resin with better UV aging resistance. However, excessively high aluminum powder content negatively affected composite strength. Thus, 6 wt.% was identified as the optimal aluminum powder content for achieving a composite material with high strength and good resistance to UV light.

It is well known that when a resin is exposed to high-energy UV radiation, the unsaturated groups in the matrix resin absorb the energy of the UV photons, which enables the transition of electrons from the ground state to the excited state. During the molecule transition from the excited state to the ground state, some free radicals were generated. These generated free radicals react with the matrix molecules, which alters the original resin molecular structure and negatively affects material properties. However, the aluminum powder incorporated in the prepared composite resins effectively reflected some of the UV light from the environment. This significantly inhibited the negative effect of UV light on the matrix resin, reduced the generation of free radicals, and inhibited the molecular bond changes caused by free radical reactions. Higher aluminum powder content in the composite material led to lower UV light absorption on the surface of the epoxy resin sample. In turn, this led to better UV aging resistance.

### 3.2. Yellowing Value

**Figure 4:**
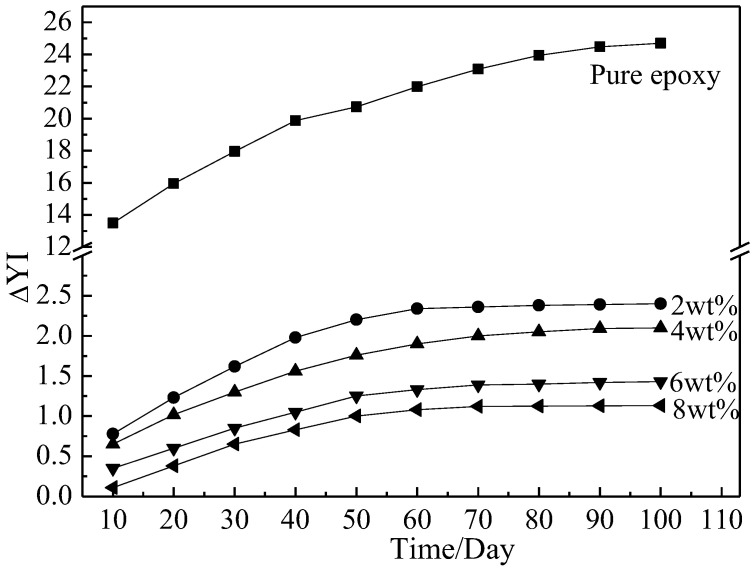
The changes to ΔYI over time of the pure epoxy and composites.

In addition to reducing polymer strength, UV light irradiation changed the apparent color of the composite samples. Moreover, the degree of color change increased with increasing irradiation time. Therefore, the yellowing value ΔYI of the composite surfaces was measured every 10 days during the aluminum powder/epoxy composite UV aging process to characterize their material surface yellowing. Figure 4 shows the trend in ΔYI over time.

**Figure 5:**
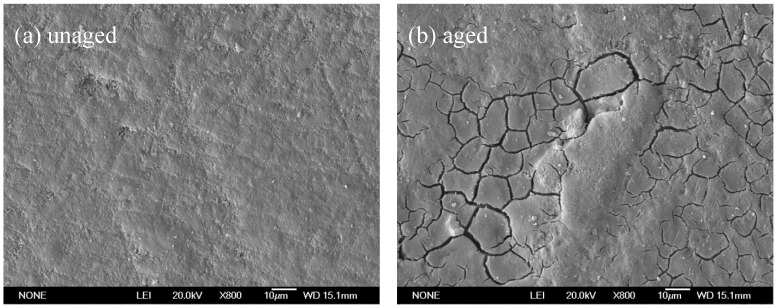
The SEM photograph of the surface of the composite coating with 6 wt.% spherical aluminum powder before and after 100 days UV irradiation: (**a**) unaged composite, (**b**) aged composite.

The composites all exhibited positive ΔYI values, and these values gradually increased with increasing irradiation time. This indicated that the surface color of the composite samples shifted to yellow and that the resin was increasingly yellowing with increasing UV radiation exposure. However, compared with the pure epoxy resin, the ΔYI values of the composite materials were significantly lower. Moreover, ΔYI decreased with increasing aluminum powder content. Thus, adding aluminum powder to the epoxy resin effectively alleviated resin yellowing under UV irradiation, and higher aluminum powder content led to a stronger protective effect. The ΔYI values of the composites with varying spherical aluminum powder content showed the same trend over time. In the first 60 days of UV irradiation, the ΔYI values of the composites significantly increased. In contrast, the ΔYI values did not significantly change during the 60–100-day irradiation period. After 100 days of UV irradiation, the ΔYI values of the composites with 2 wt.%, 4 wt.%, 6 wt.%, and 8 wt.% spherical aluminum powder content were +2.40, +2.11, +1.43, and +1.13, respectively. This trend was the same as that exhibited by the tensile strengths of the composites. This further verified that aluminum powder effectively reflected ultraviolet light in the environment, which reduced UV light absorption by the composite materials and alleviated the performance degradation caused by ultraviolet light. Figure 5 shows an SEM photograph of the surface of the composite coating with 6 wt.% spherical aluminum powder before and after 100 days UV irradiation. There were a number of cracks observed on the composite after 100 days UV irradiation. It shows that 100 days of UV irradiation was sufficient to cause severe aging of the composite.

### 3.3. EIS Analysis

**Figure 6:**
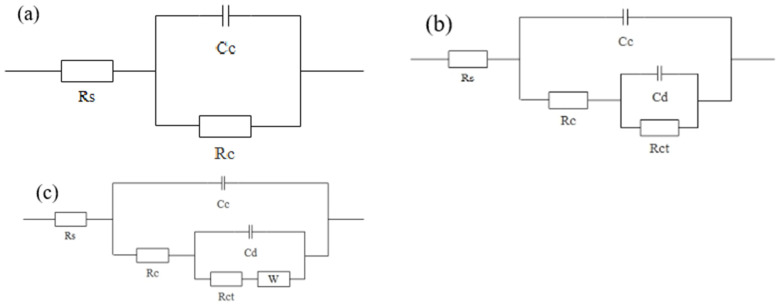
Equivalent circuit diagrams of different immersion stages: (**a**) early stage; (**b**) middle stage; (**c**) late stage.

According to the tensile strength, strength loss rate, and ∆Y values of the composites, the best strength and UV aging resistance were achieved with 6 wt.% spherical aluminum. Therefore, the composite prepared with 6 wt.% spherical aluminum was selected for EIS analysis to further investigate the impact of UV light on the protective properties of the aluminum powder/epoxy resin coating. Figure 6 shows the equivalent circuit diagram of different immersion stages, where *Rs* is the solution resistance, *Cc* is the coating capacitor, *Cd* is the solution double-layer capacitance, and *Rct* is the coating resistance. The impedance mode value |Z| at 0.01 Hz is used to characterize the barrier performance of the coating [22,23,24].

**Figure 7:**
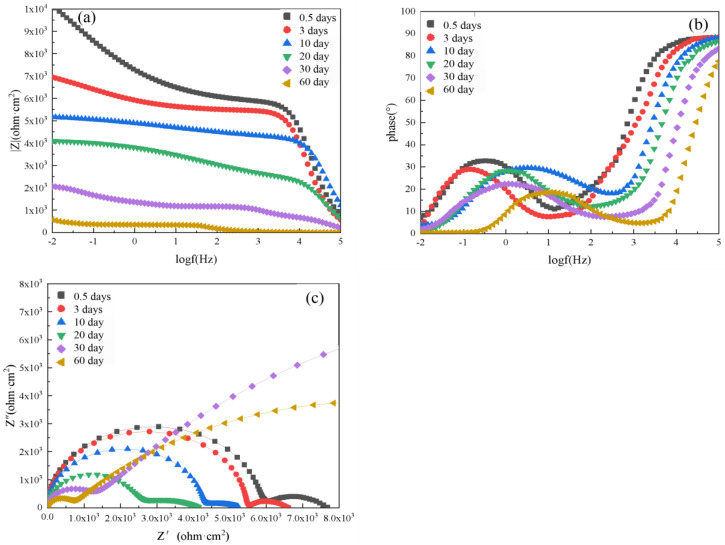
EIS results of unaged pure epoxy resin coating after immersion for various lengths of time in 3.5 wt.% NaCl solution: (**a**) Bode diagram, (**b**) phase angle diagram, and (**c**) Nyquist diagram.

The EIS results of the pure epoxy resin coating at varying immersion times are shown in Figure 7, including the Bode plot, phase angle plot, and Nyquist plot. When the pure epoxy coating was immersed in 3.5 wt.% NaCl solution for just 0.5 days, two time constants were identified, corresponding to a low-frequency impedance mode value |Z|_0.01_ of about 10,188.33 Ω·cm^2^. The phase angle of the pure epoxy coating was close to 90° within a frequency, and the maximum value was the manifestation of the second time constant. This indicated that the corrosive media had begun to penetrate into the coating and reach the metal surface, which damaged the bonding between the metal substrate and the coating. The Nyquist plots consisted of two semicircles, indicating that the coating at this time had certain barrier properties. The coating was able to protect the metal substrate from corrosive particles in the solution.

With increasing immersion time, the |Z|_0.01_ value and phase angle of the pure epoxy coating were reduced, and the radius of the Nyquist plot semicircle also decreased. After 3 days of immersion, the value of |Z|_0.01_ reached 6952 Ω·cm^2^. After 10 days, |Z|_0.01_ reached 5167.94 Ω·cm^2^, and after 20 days, |Z|_0.01_ was reduced to 4099.70 Ω·cm^2^. After 30 days, |Z|_0.01_ was about 2063.00 Ω·cm^2^, and the Nyquist plot showed a straight line with a slope of approximately 1 in the low-frequency region. This indicated that diffusion occurred at this time. Therefore, after 30 days, the corrosion medium contacted the Q235 steel substrate, which began to corrode. When the immersion time was increased to 60 days, the value of |Z|_0.01_ dropped to 579 Ω·cm^2^, which was a decrease of about two orders of magnitude compared to the value of the sample immersed for 0.5 days. This showed that the protective effect of the epoxy resin coating on the substrate significantly decreased. After 60 days, this pure epoxy coating lost most of its protective ability.

**Figure 8:**
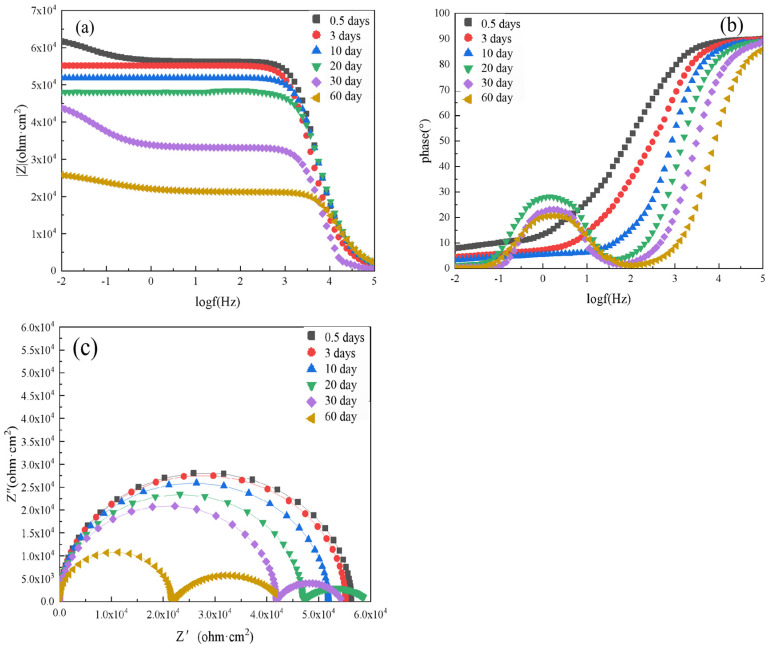
EIS results of unaged composite coating prepared with 6 wt.% spherical aluminum powder after immersion for various lengths of time in 3.5 wt.% NaCl solution: (**a**) Bode diagram, (**b**) phase angle diagram, and (**c**) Nyquist diagram.

The EIS results of the composite coating with 6 wt.% spherical aluminum powder are shown in Figure 8. When this coating was immersed in the 3.5 wt.% NaCl solution for 0.5 days, the Nyquist plot showed a semicircle. The Nyquist and phase angle plots of this composite coating demonstrated that it only had one time constant. The |Z|_0.01_ value of this coating was 61,746.48 Ω·cm^2^ in the low-frequency region, which was about 6 times higher than that of the pure epoxy resin after 0.5 days of immersion. Moreover, the phase angle was close to 90° across a very wide frequency range, which showed that the composite coating had good protective properties and could effectively prevent the corrosive medium from penetrating through the coating to the metal substrate surface.

As the immersion time of the composite coating was extended to 3 days and 10 days, its |Z|_0.01_ value gradually decreased to 55,168 Ω·cm^2^ and 51,842 Ω·cm^2^, respectively. When the composite coating was immersed for 20 days, a maximum value was observed in the phase angle plot, and the Nyquist plot showed two semicircular arcs. This indicated the appearance of the second time constant and that the corrosive media had reached the surface of the metal substrate through the coating at this time. Thus, the bonding between the substrate and the coating was damaged. At this time, the |Z|_0.01_ value of the composite coating was 48,060.27 Ω·cm^2^. After 30 days, the value of |Z|_0.01_ was 43,806.00 Ω·cm^2^; After 60 days, the value of |Z|_0.01_ was 25,791.33 Ω·cm^2^, demonstrating a significant decrease compared to 0.5 days of immersion. This showed that the protective effect of the composite coating has decreased with the longer immersion.

Compared with the pure epoxy coating, the composite coating prepared with 6 wt.% spherical aluminum powder provided better protection for the metal substrate during the NaCl immersion process. This composite coating effectively blocked corrosion medium penetration and inhibited substrate corrosion. However, with the extension of the immersion time, the protective performance of the composite coating did eventually decline.

**Figure 9:**
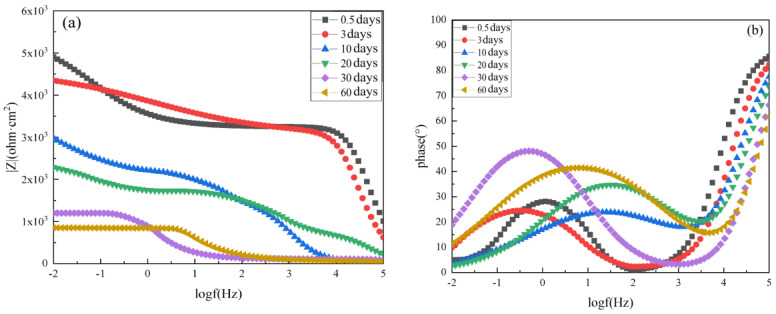

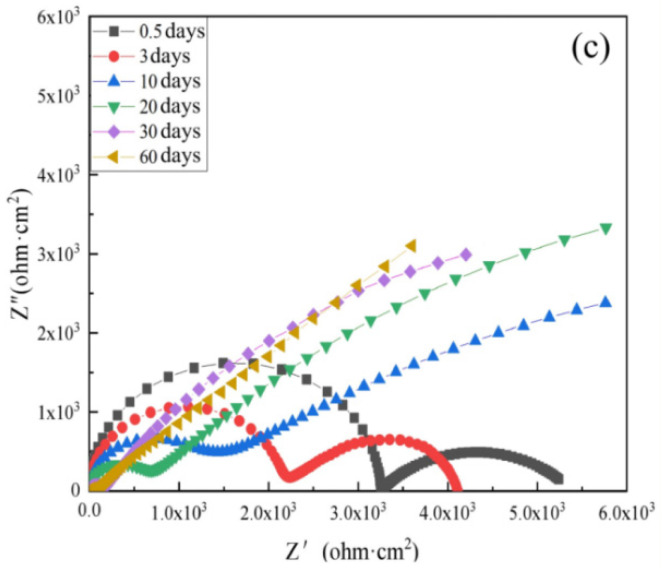
EIS results of pure epoxy resin coating after UV aging for 100 days and immersion for various lengths of time in 3.5 wt.% NaCl solution: (**a**) Bode diagram, (**b**) phase angle diagram, and (**c**) Nyquist diagram.

The EIS results of the pure epoxy resin coating after 100 days of UV aging followed by immersion in 3.5 wt.% NaCl are shown in Figure 9. The phase angle and Nyquist plots exhibited two time constants at different immersion times. When the coating was immersed in solution for 0.5 days, its |Z|_0.01_ value was 4902.13 Ω·cm^2^, which was 0.5 times that of the unaged coating after 0.5 days of immersion. This indicated that UV light aging significantly reduced the barrier properties of this pure epoxy coating. At this time, the coating resistance obtained by fitting the Nyquist plot was 3044 Ω·cm^2^, which was also much lower than that of the unaged coating immersed for 0.5 days. After 3 days of immersion, the radius of the Nyquist semicircular arc decreased, the |Z|_0.01_ value of the pure epoxy coating was 4346.77 Ω·cm^2^, and the fitted coating resistance was 2110 Ω·cm^2^. After 10 days of immersion, the Nyquist plot showed a straight line with a slope of approximately 1 in the low-frequency region, indicating a diffusion phenomenon. This showed that the corrosion media significantly penetrated the coating, reaching the surface of the metal substrate and causing metal corrosion. At this time, the |Z|_0.01_ value of the pure epoxy coating was 2982.33 Ω·cm^2^, and the coating resistance value obtained by fitting was 1142 Ω·cm^2^. After 20 days of immersion, the radius of the Nyquist semicircular arc was further reduced. This indicated that corrosion continued to intensify. Meanwhile, the |Z|_0.01_ value was 2288.59 Ω·cm^2^, and the coating resistance value obtained by fitting was 620.4 Ω·cm^2^. After 30 days of immersion, the |Z|_0.01_ value was 1200.60 Ω·cm^2^, and the coating resistance value obtained by fitting was 108.6 Ω·cm^2^. This was significantly lower than that of the coating after 0.5 days of immersion, which indicated that the pure epoxy coating lost most of its protective effect at this time. After 60 days of immersion, the |Z|_0.01_ value was 852.12 Ω·cm^2^, and the coating resistance value obtained by fitting was 58.72 Ω·cm^2^. At this time, the pure epoxy coating completely lost its protective effect, and the metal substrate was completely corroded.

**Figure 10:**
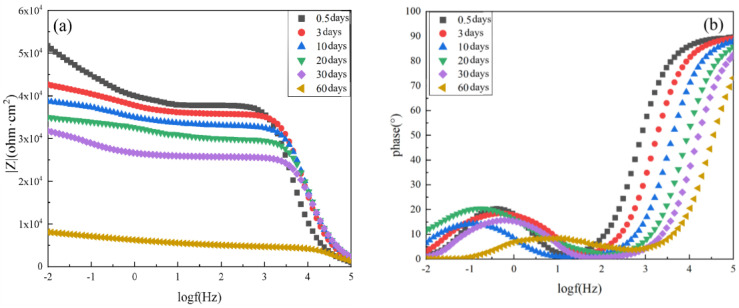

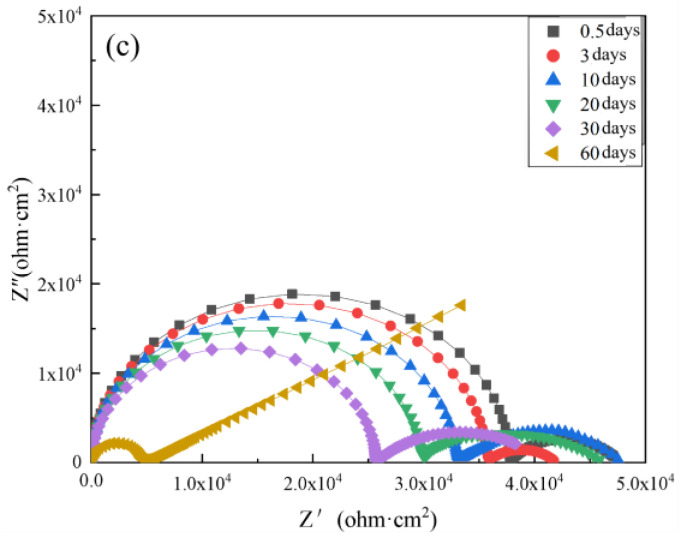
EIS results of composite coating prepared with 6 wt.% spherical aluminum powder after UV aging for 100 days and immersion for various lengths of time in 3.5 wt.% NaCl solution: (**a**) Bode diagram, (**b**) phase angle diagram, and (**c**) Nyquist diagram.

The EIS results of the composite coating prepared with 6 wt.% spherical aluminum powder after 100 days of UV aging followed by immersion in 3.5 wt.% NaCl solution are shown in Figure 10. During the initial stage of immersion, the Nyquist plot presented two semicircles of different sizes. Combined with the phase angle plot, this showed that the composite coating had two time constants during this period. The |Z|_0.01_ value at this time was 51,690.12 Ω·cm^2^, and the coating resistance value obtained by fitting was 3773 Ω·cm^2^. After 3 days of immersion, the |Z|_0.01_ value was reduced to 42,650 Ω·cm^2^, and the coating resistance value obtained by fitting was 35,660 Ω·cm^2^. After 10 days of immersion, the |Z|_0.01_ value was further reduced to 38,700.52 Ω·cm^2^, and the coating resistance value obtained by fitting was 32,754 Ω·cm^2^. After 20 days of immersion, |Z|_0.01_ further declined to 34,950.85 Ω·cm^2^, and the coating resistance was 29,639 Ω·cm^2^. After 30 days of immersion, the |Z|_0.01_ value was 31,750.08 Ω·cm^2^ and the coating resistance was 25,630 Ω·cm^2^. When the immersion time reached 60 days, a straight line with a slope of approximately 1 was visible in the low-frequency region, which indicated that a large amount of the corrosion media penetrated the coating, reached the surface of the metal substrate, and caused metal corrosion. At this time, the |Z|_0.01_ value decreased to 8103.27 ohms·cm^2^, which was lower by an order of magnitude than that of the unaged composite coating immersed for 0.5 days.

**Figure 11:**
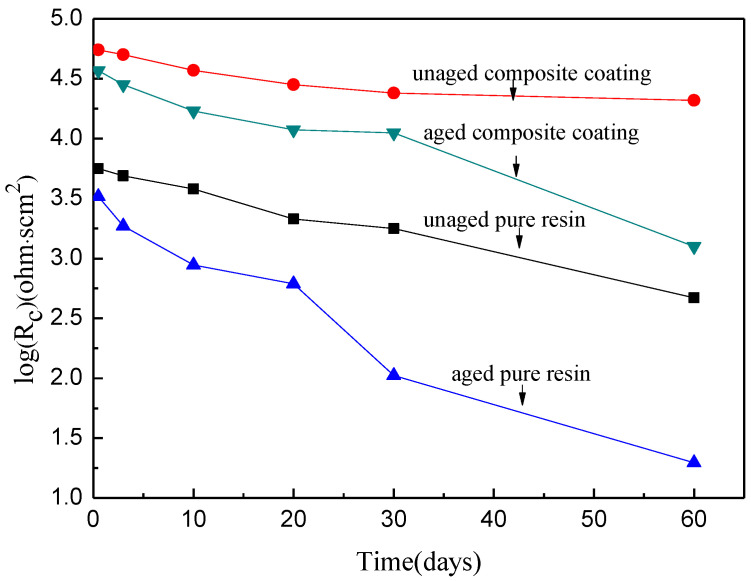
The coating resistance variation of pure epoxy resin and composite with time.

**Figure 12:**
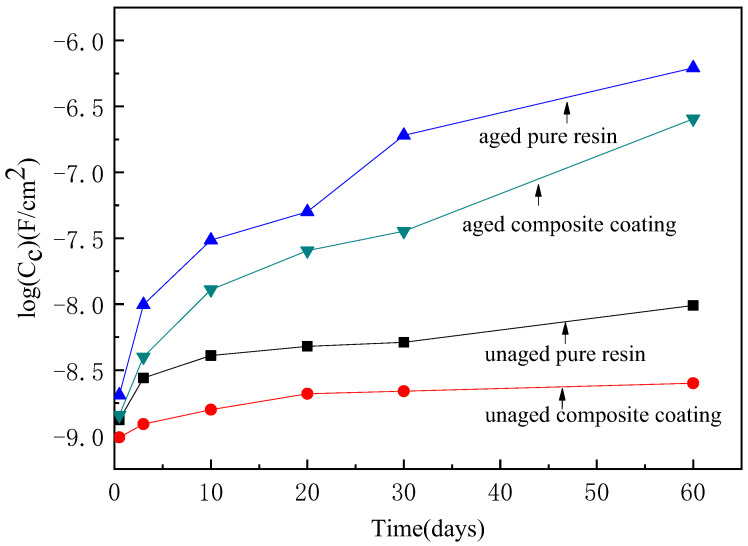
The coating capacitance variation of pure epoxy resin and composite with time.

The coating resistance and the coating capacitance of the pure epoxy coating and the composite coating prepared with 6 wt.% spherical aluminum powder before and after UV aging are shown in Figure 11 and Figure 12. Figure 11 shows that the coating resistance decreased with increasing immersion time. However, the coating resistance of the unaged composite coating was much higher than that of the unaged pure epoxy coating at equivalent immersion times. After UV aging for 100 days, the coating resistance of both the pure epoxy coating and composite coating declined, but that of the aged composite coating was still higher than that of the aged pure epoxy coating. Figure 12 shows that the coating capacitance increased with increasing immersion time. The capacitance value of the unaged composite coating was lower than that of unaged pure epoxy coating. This was also true after UV aging.

According to Figure 11 and Figure 12, the coating immersion process can be divided into four stages. In the first stage, the coating has high resistance and low capacitance. This stage is the initial period when the electrolyte enters the coating. However, with increasing immersion time, the coating capacitance gradually increases and the coating resistance gradually decreases. This stage is the second stage of the immersion process. During this second stage, water molecules penetrate the interior of the coating, meaning that this insulating coating now has a degree of electrical conductivity. A greater coating resistance means that the coating is a better barrier to water, oxygen, and other electrolytes. When the coating becomes saturated by water adsorption, it enters the third stage of the immersion process. At this time, because the coating is saturated with water molecules, its resistance value and capacitance value tend to be a certain value. With a further extension of immersion time, the corrosive particles in the electrolyte successfully penetrate through the coating and reach the metal surface. At the surface, they react with the metal at the interface between the coating and the metal surface, leading to metal corrosion. The generated corrosion products reduce the bonding force between the coating and the metal substrate. At this time, the coating resistance sharply drops and the capacitor value sharply increases. This is the fourth stage.

Comparing the unaged pure epoxy coating and the unaged composite coating shows that the pure epoxy coating reached the second immersion stage after 0.5 days of immersion. This coating then rapidly saturated in the third stage. On the 30th day of immersion, the corrosion medium penetrated the coating and reached the metal surface, causing the coating to corrode. Consequently, the change in the resistance and capacitance of the pure epoxy coating was almost a straight line. No obvious characteristics of the third immersion phase were observed in these plots. For the composite coating with the aluminum powder, the characteristics of the first, second, and third stages were clear within 60 days of immersion, but the characteristics of the fourth stage were not visible. This was because the addition of aluminum powder extended the pathway of the corrosion media into the composite coating, which enhanced the protective effect of the coating on the metal and prolonged its service life.

After UV aging, the rate of change of the resistance and capacitance values of the pure epoxy coating and the composite coating were both greater than those of the unaged coatings. This was because the UV light destroyed the three-dimensional network structure of the epoxy resin, leading to an increased number of holes and cracks in the coating. These holes and cracks enhanced the penetration of the corrosion medium through the coating to the surface of the metal substrate. Therefore, the penetration rate of the corrosion medium was significantly higher after UV aging, leading to higher coating resistance and capacitance change rates. However, the addition of aluminum powder significantly reduced the change rate of the coating resistance and capacitance caused by UV light aging. This shows that the addition of aluminum powder effectively improved the protective performance of the coating after long-term UV light illumination. This was because the aluminum powder effectively reflected the UV light, which reduced the UV light absorbed by the coating and protected the epoxy resin from UV light damage. In addition, the aluminum powder also blocked the penetration of the corrosion media. Moreover, the composite coating still maintained a good protective performance after 100 days of UV light illumination.

## 4. Conclusions

In this work, an aluminum powder/epoxy composite coating was prepared by incorporating the spherical aluminum powder in an epoxy resin. The addition of aluminum powder enhanced the strength of the composite coating and reflected UV light from the environment. This improved the resistance of the coating to UV aging, which prolonged its service life. The highest tensile strength value of the unaged composite coating was 64.5 MPa, which was achieved by using 6 wt.% aluminum powder. This was 1.2 times higher than that of the pure resin. After 100 days of accelerated UV aging, the strength of the composite decreased. The composite prepared with 6 wt.% aluminum powder displayed the highest tensile strength of 58.5 MPa after UV aging. This represented a strength loss rate of 9.3%. The yellowing value of this composite after 100 days of UV aging was +1.43. EIS testing showed that the addition of aluminum powder effectively improved the barrier property of the epoxy resin toward corrosion particles in the environment. After long-term UV aging, the protective performance of the composite coating deteriorated. However, the degree of deterioration was much lower than that of the pure epoxy resin.

## Figures and Tables

**Table 1:** Chemical composition of Q235 steel.

Chemical Composition	C	Mn	S	P	Si	Fe
Mass fraction (wt.%)	0.220	0.480	0.022	0.012	<0.050	Margin

**Table 2:** Tensile strength loss rate of pure epoxy and composites after UV aging.

	Specimen	Pure Epoxy	Composite			
Performance			2 wt.%	4 wt.%	6 wt.%	8 wt.%
Tensile strength loss rate (%)		22.7	11.5	10.4	9.3	9.0

## Data Availability

The data presented in this study are included within the article.
